# Supplementary material for: Genome-wide patterns of population structure and association mapping of nut-related traits in Persian walnut populations from Iran using the Axiom J. regia 700K SNP array
Source: Sci Rep. 2019 Apr 23;9:6376. doi: 10.1038/s41598-019-42940-1 (PMC6478883; doi:10.1038/s41598-019-42940-1)
Supplement: Supplementary file 1 — Genome-wide patterns of population structure and association mapping of nut-related traits in Persian walnut populations from Iran using the Axiom J. regia 700K SNP array [file 41598_2019_42940_MOESM1_ESM.docx]

**Genome-wide patterns of population structure and association mapping of nut-related traits in Persian walnut populations from Iran using the Axiom *J. regia*700K SNP array**

 Mohammad Mehdi Arab^1^, Annarita Marrano^2^, [Rostam Abdollahi-Arpanahi](http://scholar.google.com/citations?user=OmDA_YQAAAAJ&hl=en)^3^, Charles A. Leslie^2^, Hossein Askari^4^, David B. Neale^2^ & [Kourosh Vahdati](http://scholar.google.com/citations?user=ZPuaA8kAAAAJ&hl=en)^1^

^1^Department of Horticulture, College of Aburaihan, University of Tehran, Tehran, Iran

^2^Department of Plant Sciences, University of California, Davis, CA 95616

^3^Department of Animal and Poultry Science, College of Aburaihan, University of Tehran, Tehran, Iran

^4^ Department of Plant Sciences and Biotechnology, Faculty of Life Sciences and Biotechnology, Shahid Beheshti University, Tehran, Iran

Corresponding Authors: Kourosh Vahdati (email: kvahdati@ut.ac.ir)

**SUPPLEMENTARY INFORMATION**

**Supplementary Figures**


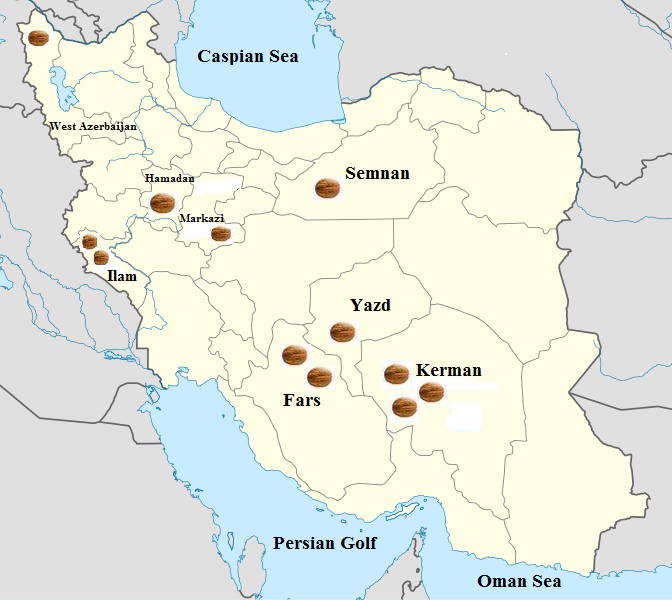


**Fig. S1.** Geographical location of collection regions of studied Iranian walnut genotypes (Sample collection areas are shown with walnut seed)

**
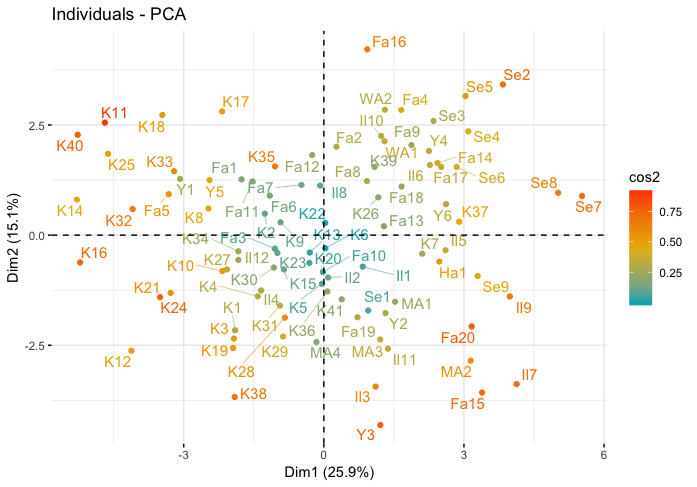
**

**Fig. S2.** Scatter plot for the first two principal components for the studied walnut genotypes based on nut and kernel traits.

**
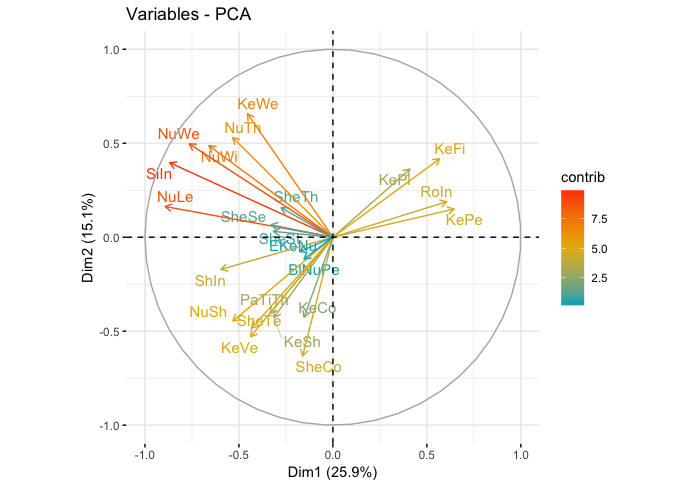
**

**Fig. S3.** Principal component analysis biplot of nuts and kernel traits of 95 Iranian walnut genotype.


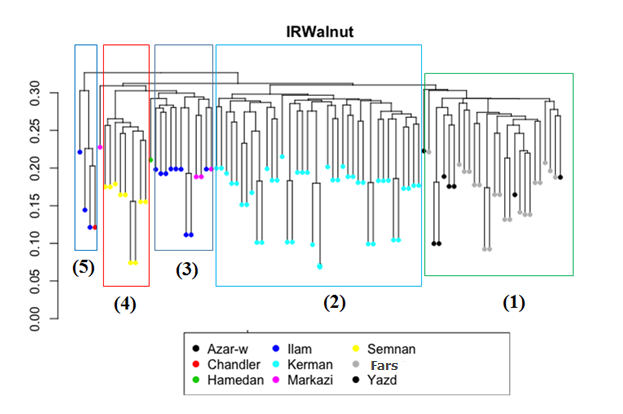


**Fig. S4.** Clusters of individuals using a specified dendrogram from hierarchical cluster analysis on the dissimilarity matrix. Each point represents an individually genotyped walnut. The samples are color‐coded by collection province.


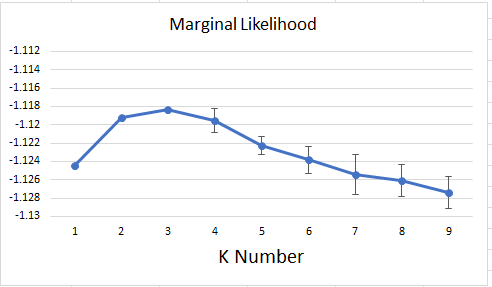


**Fig. S5.** Population structure results using 33,336 SNPs. The plateau of the graph at k = 3 indicates the minimum number of subgroups possible in the diversity panel.

| **Table S1.** The information of 95 walnut genotype analyzed | | | | | |
| --- | --- | --- | --- | --- | --- |
| Number | Province | Region | Individual ID | Population ID | Type of Material/ Sampling Site |
| 1 | Kerman | Baft-Gugher | KBG1 | K1 | Very old walnut trees from open pollinated seedlings (more than 500-year-old)/ valley |
| 2 | Kerman | Baft-Gugher | KBG2 | K2 | Very old walnut trees from open pollinated seedlings (more than 500-year-old)/ valley |
| 3 | Kerman | Baft-Gugher | KBG3 | K3 | old walnut trees from open pollinated seedlings (150- to 250-year-old)/ valley |
| 4 | Kerman | Baft-Gugher | KBG4 | K4 | old walnut trees from open pollinated seedlings (150- to 250-year-old)/ valley |
| 5 | Kerman | Baft-Gugher | KBG5 | K5 | old walnut trees from open pollinated seedlings (150- to 250-year-old)/ valley |
| 6 | Kerman | Baft-Gugher | KBG6 | K6 | old walnut trees from open pollinated seedlings (150- to 250-year-old)/ valley |
| 7 | Kerman | Baft-Gugher | KBG7 | K7 | old walnut trees from open pollinated seedlings (150- to 250-year-old)/ valley |
| 8 | Kerman | Baft-Gugher | KBG8 | K8 | old walnut trees from open pollinated seedlings (150- to 250-year-old)/ valley |
| 9 | Kerman | Baft-Gugher | KBG9 | K9 | Very old walnut trees from open pollinated seedlings (more than 500-year-old)/ valley |
| 10 | Kerman | Baft-Gugher | KBG10 | K10 | old walnut trees from open pollinated seedlings (150- to 250-year-old)/ valley |
| 11 | Kerman | Baft-Gugher | KBG11 | K11 | Very old walnut trees from open pollinated seedlings (more than 500-year-old)/ valley |
| 12 | Kerman | Baft-Gugher | KBG12 | K12 | Very old walnut trees from open pollinated seedlings (more than 500-year-old)/ valley |
| 13 | Kerman | Baft-Gugher | KBG13 | K13 | old walnut trees from open pollinated seedlings (150- to 250-year-old)/ valley |
| 14 | Kerman | Rabor | KR1 | K14 | old walnut trees from open pollinated seedlings (100- to 200-year-old)/ valley |
| 15 | Kerman | Rabor | KR2 | K15 | old walnut trees from open pollinated seedlings (100- to 200-year-old)/ valley |
| 16 | Kerman | Rabor | KR3 | K16 | old walnut trees from open pollinated seedlings (100- to 200-year-old)/ valley |
| 17 | Kerman | Rabor | KR4 | K17 | old walnut trees from open pollinated seedlings (100- to 200-year-old)/ valley |
| 18 | Kerman | Rabor | KR5 | K18 | old walnut trees from open pollinated seedlings (100- to 200-year-old)/ valley |
| 19 | Kerman | Rabor | KR6 | K19 | old walnut trees from open pollinated seedlings (100- to 200-year-old)/ valley |
| 20 | Kerman | Rabor | KR7 | K20 | old walnut trees from open pollinated seedlings (100- to 200-year-old)/ valley |
| 21 | Kerman | Rabor | KR8 | K21 | old walnut trees from open pollinated seedlings (100- to 200-year-old)/ valley |
| 22 | Kerman | Rabor | KR9 | K22 | old walnut trees from open pollinated seedlings (100- to 200-year-old)/ valley |
| 23 | Kerman | Rabor | KR10 | K23 | old walnut trees from open pollinated seedlings (100- to 200-year-old)/ valley |
| 24 | Kerman | Rabor | KR11 | K24 | old walnut trees from open pollinated seedlings (100- to 200-year-old)/ valley |
| 25 | Kerman | Rabor | KR12 | K25 | old walnut trees from open pollinated seedlings (100- to 200-year-old)/ valley |
| 26 | Kerman | Rabor | KR13 | K26 | old walnut trees from open pollinated seedlings (100- to 200-year-old)/ valley |
| 27 | Kerman | Rabor | KR14 | K27 | old walnut trees from open pollinated seedlings (100- to 200-year-old)/ valley |
| 28 | Kerman | Rabor | KR15 | K28 | old walnut trees from open pollinated seedlings (100- to 200-year-old)/ valley |
| 29 | Kerman | Rabor | KR16 | K29 | old walnut trees from open pollinated seedlings (100- to 200-year-old)/ valley |
| 30 | Kerman | Rabor-Hanza | KRH1 | K30 | old walnut trees from open pollinated seedlings (150- to 200-year-old)/ valley |
| 31 | Kerman | Rabor-Hanza | KRH2 | K31 | old walnut trees from open pollinated seedlings (150- to 200-year-old)/ valley |
| 32 | Kerman | Rabor-Hanza | KRH3 | K32 | old walnut trees from open pollinated seedlings (150- to 200-year-old)/ valley |
| 33 | Kerman | Rabor-Hanza | KRH4 | K33 | old walnut trees from open pollinated seedlings (150- to 200-year-old)/ valley |
| 34 | Kerman | Rabor-Hanza | KRH5 | K34 | old walnut trees from open pollinated seedlings (150- to 200-year-old)/ valley |
| 35 | Kerman | Rabor-Hanza | KRH6 | K35 | old walnut trees from open pollinated seedlings (150- to 200-year-old)/ valley |
| 36 | Kerman | Bardsir | KB1 | K36 | old walnut trees from open pollinated seedlings (80- to 150-year-old)/ valley |
| 37 | Kerman | Bardsir | KB2 | K37 | old walnut trees from open pollinated seedlings (80- to 150-year-old)/ valley |
| 38 | Kerman | Bardsir | KB3 | K38 | old walnut trees from open pollinated seedlings (80- to 150-year-old)/ valley |
| 39 | Kerman | Bardsir | KB4 | K39 | old walnut trees from open pollinated seedlings (80- to 150-year-old)/ valley |
| 40 | Kerman | Bardsir | KB5 | K40 | old walnut trees from open pollinated seedlings (80- to 150-year-old)/ valley |
| 41 | Kerman | Bardsir | KB6 | K41 | old walnut trees from open pollinated seedlings (80- to 150-year-old)/ valley |
| 42 | Fars | Eqlid | FaEq1 | Fa1 | old walnut trees from open pollinated seedlings (50- to 100-year-old)/ mountainous areas |
| 43 | Fars | Eqlid | FaEq2 | Fa2 | old walnut trees from open pollinated seedlings (50- to 100-year-old)/ mountainous areas |
| 44 | Fars | Eqlid | FaEq3 | Fa3 | old walnut trees from open pollinated seedlings (50- to 100-year-old)/ mountainous areas |
| 45 | Fars | Eqlid | FaEq4 | Fa4 | old walnut trees from open pollinated seedlings (50- to 100-year-old)/ mountainous areas |
| 46 | Fars | Eqlid | FaEq5 | Fa5 | old walnut trees from open pollinated seedlings (50- to 100-year-old)/ mountainous areas |
| 47 | Fars | Eqlid | FaEq6 | Fa6 | old walnut trees from open pollinated seedlings (50- to 100-year-old)/ mountainous areas |
| 48 | Fars | Eqlid | FaEq7 | Fa7 | old walnut trees from open pollinated seedlings (50- to 100-year-old)/ mountainous areas |
| 49 | Fars | Eqlid | FaEq8 | Fa8 | old walnut trees from open pollinated seedlings (50- to 100-year-old)/ mountainous areas |
| 50 | Fars | Eqlid | FaEq9 | Fa9 | old walnut trees from open pollinated seedlings (50- to 100-year-old)/ mountainous areas |
| 51 | Fars | Eqlid | FaEq10 | Fa10 | old walnut trees from open pollinated seedlings (50- to 100-year-old)/ mountainous areas |
| 52 | Fars | Eqlid | FaEq11 | Fa11 | old walnut trees from open pollinated seedlings (50- to 100-year-old)/ mountainous areas |
| 53 | Fars | Eqlid | FaEq12 | Fa12 | old walnut trees from open pollinated seedlings (50- to 100-year-old)/ mountainous areas |
| 54 | Fars | Eqlid | FaEq13 | Fa13 | old walnut trees from open pollinated seedlings (50- to 100-year-old)/ mountainous areas |
| 55 | Fars | Eqlid | FaEq14 | Fa14 | old walnut trees from open pollinated seedlings (50- to 100-year-old)/ mountainous areas |
| 56 | Fars | Eqlid | FaEq15 | Fa15 | old walnut trees from open pollinated seedlings (50- to 100-year-old)/ mountainous areas |
| 57 | Fars | Bavanat | FaBa1 | Fa16 | old walnut trees from open pollinated seedlings (50- to 100-year-old)/ mountainous areas |
| 58 | Fars | Bavanat | FaBa2 | Fa17 | old walnut trees from open pollinated seedlings (50- to 100-year-old)/ mountainous areas |
| 59 | Fars | Bavanat | FaBa3 | Fa18 | old walnut trees from open pollinated seedlings (50- to 100-year-old)/ mountainous areas |
| 60 | Fars | Bavanat | FaBa4 | Fa19 | old walnut trees from open pollinated seedlings (50- to 100-year-old)/ mountainous areas |
| 61 | Fars | Bavanat | FaBa5 | Fa20 | old walnut trees from open pollinated seedlings (50- to 100-year-old)/ mountainous areas |
| 62 | Semnan | Shahmirzad | SeSh1 | Se1 | old walnut trees from open pollinated seedlings (80- to 150-year-old)/ mountainous areas |
| 63 | Semnan | Shahmirzad | SeSh2 | Se2 | old walnut trees from open pollinated seedlings (80- to 150-year-old)/ mountainous areas |
| 64 | Semnan | Shahmirzad | SeSh3 | Se3 | old walnut trees from open pollinated seedlings (80- to 150-year-old)/ mountainous areas |
| 65 | Semnan | Shahmirzad | SeSh4 | Se4 | old walnut trees from open pollinated seedlings (80- to 150-year-old)/ mountainous areas |
| 66 | Semnan | Shahmirzad | SeSh5 | Se5 | old walnut trees from open pollinated seedlings (80- to 150-year-old)/ mountainous areas |
| 67 | Semnan | Shahmirzad | SeSh6 | Se6 | old walnut trees from open pollinated seedlings (80- to 150-year-old)/ mountainous areas |
| 68 | Semnan | Shahmirzad | SeSh7 | Se7 | old walnut trees from open pollinated seedlings (80- to 150-year-old)/ mountainous areas |
| 69 | Semnan | Shahmirzad | SeSh8 | Se8 | old walnut trees from open pollinated seedlings (80- to 150-year-old)/ mountainous areas |
| 70 | Semnan | Shahmirzad | SeSh9 | Se9 | old walnut trees from open pollinated seedlings (80- to 150-year-old)/ mountainous areas |
| 71 | Ilam | Ilam | IlIl1 | Il1 | old walnut trees from open pollinated seedlings (50- to 100-year-old)/ mountainous areas |
| 72 | Ilam | Ilam | IlIl2 | Il2 | old walnut trees from open pollinated seedlings (50- to 100-year-old)/ mountainous areas |
| 73 | Ilam | Ilam | IlIl3 | Il3 | old walnut trees from open pollinated seedlings (50- to 100-year-old)/ mountainous areas |
| 74 | Ilam | Ilam | IlIl4 | Il4 | old walnut trees from open pollinated seedlings (50- to 100-year-old)/ mountainous areas |
| 75 | Ilam | Ilam | IlIl5 | Il5 | old walnut trees from open pollinated seedlings (50- to 100-year-old)/ mountainous areas |
| 76 | Ilam | Ilam | IlIl6 | Il6 | old walnut trees from open pollinated seedlings (50- to 100-year-old)/ mountainous areas |
| 77 | Ilam | Eyvan | IlEy1 | Il7 | old walnut trees from open pollinated seedlings (50- to 100-year-old)/ mountainous areas |
| 78 | Ilam | Eyvan | IlEy2 | Il8 | old walnut trees from open pollinated seedlings (50- to 100-year-old)/ mountainous areas |
| 78 | Ilam | Eyvan | IlEy3 | Il9 | old walnut trees from open pollinated seedlings (50- to 100-year-old)/ mountainous areas |
| 80 | Ilam | Eyvan | IlEy4 | Il10 | old walnut trees from open pollinated seedlings (50- to 100-year-old)/ mountainous areas |
| 81 | Ilam | Eyvan | IlEy5 | Il11 | old walnut trees from open pollinated seedlings (50- to 100-year-old)/ mountainous areas |
| 82 | Ilam | Eyvan | IlEy6 | Il12 | old walnut trees from open pollinated seedlings (50- to 100-year-old)/ mountainous areas |
| 83 | Yazd | Taft | YT1 | Y1 | old walnut trees from open pollinated seedlings (100- to 150-year-old)/ mountainous areas |
| 84 | Yazd | Taft | YT2 | Y2 | old walnut trees from open pollinated seedlings (100- to 150-year-old)/ mountainous areas |
| 85 | Yazd | Taft | YT3 | Y3 | old walnut trees from open pollinated seedlings (100- to 150-year-old)/ mountainous areas |
| 86 | Yazd | Taft | YT4 | Y4 | old walnut trees from open pollinated seedlings (100- to 150-year-old)/ mountainous areas |
| 87 | Yazd | Taft | YT5 | Y5 | old walnut trees from open pollinated seedlings (100- to 150-year-old)/ mountainous areas |
| 88 | Yazd | Taft | YT6 | Y6 | old walnut trees from open pollinated seedlings (100- to 150-year-old)/ mountainous areas |
| 89 | Markazi | Delijan-Jasb | MDJ1 | MA1 | old walnut trees from open pollinated seedlings (50- to 100-year-old)/ mountainous areas |
| 90 | Markazi | Delijan-Jasb | MDJ2 | MA2 | old walnut trees from open pollinated seedlings (50- to 100-year-old)/ mountainous areas |
| 91 | Markazi | Delijan-Jasb | MDJ3 | MA3 | old walnut trees from open pollinated seedlings (50- to 100-year-old)/ mountainous areas |
| 92 | Markazi | Delijan-Jasb | MDJ4 | MA4 | old walnut trees from open pollinated seedlings (50- to 100-year-old)/ mountainous areas |
| 93 | West Azerbaijan | Khoy | WAK1 | WA1 | old walnut trees from open pollinated seedlings (50- to 100-year-old)/ mountainous areas |
| 94 | West Azerbaijan | Khoy | WAK2 | WA2 | old walnut trees from open pollinated seedlings (50- to 100-year-old) |
| 95 | Hamadan | Nahavand | HaNa1 | Ha1 | old walnut trees from open pollinated seedlings (50- to 100-year-old) |

| **Table S2.** Eigenvalues of the eight principal component of fruit traits in the studied walnuts | | | | | | | | |
| --- | --- | --- | --- | --- | --- | --- | --- | --- |
| Variable | PC1 | PC2 | PC3 | PC4 | PC5 | PC6 | PC7 | PC8 |
| NuLe | 0.366 | 0.088 | 0.198 | 0.064 | -0.005 | 0.039 | 0.017 | 0.105 |
| NuWi | 0.27 | 0.262 | -0.17 | -0.261 | 0.154 | 0.014 | 0.086 | 0.1 |
| NuTh | 0.218 | 0.285 | -0.251 | -0.265 | 0.162 | -0.01 | 0.086 | 0.094 |
| NuWe | 0.313 | 0.267 | 0.013 | 0.055 | -0.012 | -0.152 | -0.135 | -0.195 |
| KePe | -0.264 | 0.081 | -0.184 | 0.289 | -0.076 | -0.039 | 0.247 | 0.009 |
| ShIn | 0.244 | -0.092 | 0.375 | 0.249 | -0.125 | 0.031 | -0.026 | 0.045 |
| SiIn | 0.356 | 0.213 | -0.011 | -0.119 | 0.091 | 0.023 | 0.061 | 0.119 |
| RoIn | -0.248 | 0.102 | -0.372 | -0.238 | 0.11 | -0.054 | 0 | -0.046 |
| NuSh | 0.218 | -0.24 | 0.172 | 0.154 | 0.129 | -0.102 | -0.182 | 0.205 |
| SheTh | 0.113 | 0.084 | -0.323 | 0.287 | -0.195 | 0.026 | -0.216 | 0.128 |
| SheCo | 0.066 | -0.34 | -0.185 | 0.042 | 0.358 | 0.01 | -0.194 | -0.136 |
| SheTe | 0.178 | -0.26 | -0.193 | 0.042 | 0.376 | 0.158 | 0.131 | 0.101 |
| SheSe | 0.135 | 0.035 | 0.045 | 0.14 | 0.096 | 0.523 | 0.464 | -0.437 |
| SheSt | 0.13 | 0.017 | -0.298 | 0.321 | -0.092 | 0.069 | -0.042 | 0.388 |
| PaTiTh | 0.137 | -0.217 | -0.156 | 0.092 | 0.314 | 0.144 | -0.211 | -0.229 |
| KeWe | 0.186 | 0.353 | -0.095 | 0.243 | -0.075 | -0.189 | 0.014 | -0.222 |
| KeCo | 0.063 | -0.229 | -0.286 | 0.083 | -0.005 | -0.234 | 0.148 | 0.198 |
| KePl | -0.168 | 0.195 | 0.086 | 0.323 | 0.256 | -0.052 | 0.271 | 0.282 |
| KeSh | 0.129 | -0.229 | -0.147 | -0.163 | -0.317 | -0.246 | 0.348 | -0.145 |
| KeVe | 0.18 | -0.285 | -0.017 | -0.072 | -0.187 | 0.059 | 0.464 | 0.234 |
| KeFi | -0.233 | 0.224 | 0.061 | 0.252 | 0.314 | 0.079 | 0.137 | 0.096 |
| EKeNu | 0.073 | -0.041 | -0.322 | 0.323 | -0.28 | 0.102 | -0.091 | -0.316 |
| BlNuPe | 0.063 | -0.062 | 0.08 | 0.167 | 0.29 | -0.679 | 0.214 | -0.283 |
| Eigenvalue | 5.9513 | 3.4632 | 2.914 | 2.335 | 1.282 | 1.0635 | 0.9809 | 0.893 |
| Proportion | 0.259 | 0.151 | 0.127 | 0.102 | 0.056 | 0.046 | 0.043 | 0.039 |
| Cumulative | 0.259 | 0.409 | 0.536 | 0.638 | 0.693 | 0.74 | 0.782 | 0.821 |

Traits abbreviations are explained in detail in the material and method section.

| **Table S3**. Ancestry values inferred by fastSTRUCTURE for 96 walnut individuals genotyped at 33,336 SNP loci. The six subgroups inferred based on a membership cutoff of 0.75 are highlighted. | | | | | | | |
| --- | --- | --- | --- | --- | --- | --- | --- |
|  |  | **Cluster membership** | | | | | |
| **Province** | Sample id | K1 | K2 | K3 | K4 | K5 | K6 |
| **Kerman** | K31 | 1 | 0 | 0 | 0 | 0 | 0 |
| **Kerman** | K33 | 1 | 0 | 0 | 0 | 0 | 0 |
| **Kerman** | K34 | 1 | 0 | 0 | 0 | 0 | 0 |
| **Kerman** | K36 | 1 | 0 | 0 | 0 | 0 | 0 |
| **Kerman** | K38 | 1 | 0 | 0 | 0 | 0 | 0 |
| **Kerman** | K40 | 1 | 0 | 0 | 0 | 0 | 0 |
| **Kerman** | K32 | 0.9995 | 0 | 0 | 0 | 0 | 0.0005 |
| **Kerman** | K14 | 0.9994 | 0 | 0 | 0.0006 | 0 | 0 |
| **Kerman** | K15 | 0.9992 | 0 | 0 | 0.0008 | 0 | 0 |
| **Kerman** | K16 | 0.9989 | 0 | 0 | 0.0011 | 0 | 0 |
| **Kerman** | K29 | 0.9971 | 0 | 0 | 0.0029 | 0 | 0 |
| **Kerman** | K30 | 0.9964 | 0 | 0 | 0.0036 | 0 | 0 |
| **Kerman** | K28 | 0.9961 | 0 | 0 | 0.0039 | 0 | 0 |
| **Kerman** | K19 | 0.9939 | 0 | 0 | 0.0061 | 0 | 0 |
| **Kerman** | K21 | 0.9904 | 0 | 0 | 0.0095 | 0 | 0 |
| **Kerman** | K17 | 0.9886 | 0 | 0 | 0.0114 | 0 | 0 |
| **Kerman** | K27 | 0.9851 | 0 | 0.0058 | 0.0091 | 0 | 0 |
| **Kerman** | K18 | 0.98 | 0 | 0 | 0.02 | 0 | 0 |
| **Kerman** | K26 | 0.9723 | 0 | 0.0184 | 0.0093 | 0 | 0 |
| **Kerman** | K20 | 0.9674 | 0 | 0 | 0.0326 | 0 | 0 |
| **Kerman** | K25 | 0.9528 | 0 | 0 | 0.0472 | 0 | 0 |
| **Kerman** | K8 | 0.9518 | 0 | 0.0039 | 0.0309 | 0 | 0.0133 |
| **Kerman** | K37 | 0.9466 | 0 | 0 | 0.0533 | 0 | 0 |
| **Kerman** | K35 | 0.938 | 0 | 0 | 0.062 | 0 | 0 |
| **Kerman** | K24 | 0.9156 | 0 | 0.0001 | 0.0843 | 0 | 0 |
| **Kerman** | K5 | 0.9002 | 0 | 0 | 0.0998 | 0 | 0 |
| **Kerman** | K13 | 0.8993 | 0.0796 | 0 | 0.0211 | 0 | 0 |
| **Kerman** | K4 | 0.883 | 0 | 0 | 0.0169 | 0 | 0.1 |
| **Kerman** | K39 | 0.8796 | 0 | 0 | 0 | 0 | 0.1204 |
| **Kerman** | K1 | 0.8733 | 0 | 0.0005 | 0.0057 | 0 | 0.1204 |
| **Kerman** | K10 | 0.8121 | 0.1 | 0.0201 | 0.0678 | 0 | 0 |
| **Kerman** | K7 | 0.7536 | 0 | 0 | 0.1463 | 0 | 0.1 |
| **Kerman** | K9 | 0.2822 | 0.7 | 0.0044 | 0 | 0 | 0.0133 |
| **Kerman** | K11 | 0.2133 | 0.7 | 0 | 0.0867 | 0 | 0 |
| **Semnan** | Se3 | 0 | 0 | 1 | 0 | 0 | 0 |
| **Semnan** | Se4 | 0 | 0 | 1 | 0 | 0 | 0 |
| **Semnan** | Se5 | 0 | 0 | 1 | 0 | 0 | 0 |
| **Semnan** | Se6 | 0 | 0 | 1 | 0 | 0 | 0 |
| **Semnan** | Se7 | 0 | 0 | 1 | 0 | 0 | 0 |
| **Semnan** | Se8 | 0 | 0 | 1 | 0 | 0 | 0 |
| **Semnan** | Se2 | 0.0001 | 0 | 0.9999 | 0 | 0 | 0 |
| **Semnan** | Se9 | 0.0018 | 0 | 0.9981 | 0 | 0 | 0 |
| **Semnan** | Se1 | 0.0035 | 0 | 0.9965 | 0 | 0 | 0 |
| **Fars** | Fa2 | 0 | 0 | 0 | 1 | 0 | 0 |
| **Fars** | Fa3 | 0 | 0 | 0 | 1 | 0 | 0 |
| **Fars** | Fa5 | 0 | 0 | 0 | 1 | 0 | 0 |
| **Fars** | Fa6 | 0 | 0 | 0 | 1 | 0 | 0 |
| **Fars** | Fa7 | 0 | 0 | 0 | 1 | 0 | 0 |
| **Fars** | Fa8 | 0 | 0 | 0 | 1 | 0 | 0 |
| **Fars** | Fa9 | 0 | 0 | 0 | 1 | 0 | 0 |
| **Fars** | Fa10 | 0 | 0 | 0 | 1 | 0 | 0 |
| **Fars** | Fa11 | 0 | 0 | 0 | 1 | 0 | 0 |
| **Fars** | Fa 13 | 0 | 0 | 0 | 1 | 0 | 0 |
| **Fars** | Fa14 | 0 | 0 | 0 | 1 | 0 | 0 |
| **Fars** | Fa15 | 0 | 0 | 0 | 1 | 0 | 0 |
| **West Azerbaijan** | WA2 | 0 | 0 | 0 | 1 | 0 | 0 |
| **Fars** | Fa4 | 0.0691 | 0 | 0 | 0.9309 | 0 | 0 |
| **Fars** | Fa12 | 0.0962 | 0 | 0 | 0.9038 | 0 | 0 |
| **Yazd** | Y4 | 0.0021 | 0 | 0 | 0.8979 | 0 | 0.1 |
| **Yazd** | Y3 | 0.0123 | 0 | 0 | 0.8877 | 0 | 0.1 |
| **Fars** | Fa20 | 0.1104 | 0 | 0.0021 | 0.8875 | 0 | 0 |
| **Yazd** | Y1 | 0.1265 | 0 | 0.0001 | 0.8729 | 0 | 0.0006 |
| **Fars** | Fa16 | 0.1324 | 0 | 0 | 0.8676 | 0 | 0 |
| **Yazd** | Y2 | 0.1335 | 0 | 0.0147 | 0.8518 | 0 | 0 |
| **Fars** | Fa17 | 0.1764 | 0 | 0 | 0.8236 | 0 | 0 |
| **Yazd** | Y6 | 0.1719 | 0 | 0.007 | 0.8211 | 0 | 0 |
| **Fars** | Fa19 | 0.2097 | 0 | 0 | 0.7903 | 0 | 0 |
| **Fars** | Fa18 | 0.2172 | 0 | 0 | 0.7828 | 0 | 0 |
| **Fars** | Fa1 | 0.2245 | 0 | 0.0059 | 0.7696 | 0 | 0 |
| **Chandler** | Ch1 | 0 | 0 | 0 | 0 | 1 | 0 |
| **Ilam** | Il9 | 0 | 0 | 0.0318 | 0 | 0.9682 | 0 |
| **Ilam** | Il8 | 0 | 0 | 0 | 0.0699 | 0.93 | 0 |
| **Kerman** | K3 | 0.5994 | 0.1 | 0 | 0.0006 | 0 | 0.3 |
| **Kerman** | K2 | 0.7 | 0 | 0 | 0 | 0 | 0.3 |
| **Kerman** | K22 | 0.7 | 0 | 0 | 0 | 0 | 0.3 |
| **Kerman** | K41 | 0.7 | 0 | 0 | 0 | 0 | 0.3 |
| **Kerman** | K23 | 0.7 | 0 | 0 | 0.0577 | 0 | 0.2423 |
| **Ilam** | Il6 | 0.1422 | 0 | 0.2812 | 0.3746 | 0.002 | 0.2 |
| **Ilam** | Il3 | 0.0748 | 0 | 0.5465 | 0.1787 | 0 | 0.2 |
| **Kerman** | K6 | 0.6811 | 0.1 | 0 | 0.0189 | 0 | 0.2 |
| **Ilam** | Il2 | 0.1409 | 0 | 0.5098 | 0.1588 | 0 | 0.1905 |
| **Markazi** | MA1 | 0.0696 | 0 | 0.238 | 0.5181 | 0 | 0.1743 |
| **Ilam** | Il7 | 0.1738 | 0 | 0.3268 | 0.3297 | 0 | 0.1697 |
| **Ilam** | Il1 | 0.2768 | 0 | 0.2859 | 0.2648 | 0.0044 | 0.1681 |
| **Ilam** | Il4 | 0.1963 | 0 | 0.2831 | 0.3564 | 0 | 0.1641 |
| **Ilam** | Il5 | 0.2192 | 0 | 0.2931 | 0.3374 | 0 | 0.1503 |
| **Hamedan** | Ha1 | 0.3372 | 0 | 0.2251 | 0.311 | 0 | 0.1268 |
| **Ilam** | Il10 | 0.3646 | 0 | 0.2373 | 0.2925 | 0 | 0.1056 |
| **Markazi** | MA2 | 0.1008 | 0 | 0.2072 | 0.6031 | 0 | 0.0889 |
| **Markazi** | MA4 | 0.1248 | 0 | 0.2111 | 0.5787 | 0 | 0.0854 |
| **Markazi** | MA3 | 0.0482 | 0 | 0.3025 | 0.5654 | 0 | 0.0839 |
| **Ilam** | Il11 | 0.1248 | 0 | 0.3238 | 0.4814 | 0 | 0.07 |
| **West Azerbaijan** | WA1 | 0.3179 | 0 | 0.0819 | 0.581 | 0 | 0.0193 |
| **Ilam** | Il12 | 0.3383 | 0 | 0.0452 | 0.1617 | 0.4538 | 0.001 |
| **Yazd** | Y5 | 0.2214 | 0 | 0.0483 | 0.7303 | 0 | 0 |
| **Kerman** | K12 | 0.3018 | 0.6394 | 0 | 0.0588 | 0 | 0 |

| **Table S4.** Global genetic differentiation statistics for all Iranian walnut populations across all loci (313,657 SNPs) | | | |
| --- | --- | --- | --- |
| stat | actual | lower | upper |
| G^′^_ST_^a^ | 0.09 | 0.05 | 0.13 |
| DJost^b^ | 0.01 | 0.00 | 0.02 |
| Fst^c^ | 0.07 | 0.06 | 0.08 |
| F_IT_^d^ | 0.11 | 0.10 | 0.12 |
| F_IS_^e^ | 0.04 | 0.03 | 0.05 |

^a^G^′^_ST_ = Hedrick’s standardized “differentiation” per locus (Hedrick, 2005); ^b^DJost = Jost’s true allelic differentiation per locus (Jost, 2008); ^c^F_ST_ = Weir and Cockerham’s fixation index estimator (Weir & Cockerham, 1984); ^d^F_IT_ = Weir and Cockerham’s overall fixation index estimator (Weir and Cockerham, 1984); ^e^F_IS_ = Weir and Cockerham’s inbreeding coefficient estimator (Weir & Cockerham, 1984)

| **Table S5.** Pairwise values of Jost’s true allelic differentiation per locus (DJost; below diagonal) and Hedrick’s standardized “differentiation” per locus (G^′^_ST_; above diagonal) between populations of Iranian walnut across all loci (313,657 SNPs) | | | | | | | |
| --- | --- | --- | --- | --- | --- | --- | --- |
|  | Kerman | Fars | Ilam | Semnan | Yazd | Markazi | West Azerbaijan |
| Kerman | - | 0.064 | 0.0547 | 0.1134 | 0.0778 | 0.0719 | 0.05 |
| Fars | 0.0077 | - | 0.063 | 0.1308 | 0.0479 | 0.0608 | 0.0093 |
| Ilam | 0.0048 | 0.0056 | - | 0.0715 | 0.0757 | 0.0361 | 0.0359 |
| Semnan | 0.0142 | 0.0163 | 0.0052 | - | 0.141 | 0.0995 | 0.1079 |
| Yazd | 0.0062 | 0.002 | 0.0046 | 0.0134 | - | 0.0779 | 0.0455 |
| Markazi | 0.0036 | 0.0022 | 0.0004 | 0.0052 | 0.0023 | - | 0.0332 |
| West Azerbaijan | 0.0002 | 0.0000 | 0.0000 | 0.0023 | 0.0000 | 0.0000 | - |

|  | **Table S6.** Annotations of significant SNPs for nut and kernel related traits. Traits abbreviations are explained in detail in the material and method section. | | | | | | | | | |
| --- | --- | --- | --- | --- | --- | --- | --- | --- | --- | --- |
| Trait | | Model | SNP ID | CHR | Position | P value | Effect | MAF | *J.regia* annotation | *E* value |
| NuWi | | SUPER | AX-170990851 | 1 | 8554385 | 2.30E-07 | -2.07 | 0.27 | None | NA |
| NuWi_BLUP | | SUPER | AX-170990851 | 1 | 8554385 | 3.00E-07 | -1.24 | 0.27 | None | NA |
| NuTh | | FarmCPU | AX-170990851 | 1 | 8554385 | 3.24E-07 | -2.33 | 0.27 | None | NA |
| NuTh_BLUP | | FarmCPU | AX-170990851 | 1 | 8554385 | 3.29E-07 | -1.70 | 0.27 | None | NA |
| NuWe | | FarmCPU | AX-170551302 | 7 | 43396444 | 4.54E-07 | -2.24 | 0.12 | None | NA |
| NuWe | | FarmCPU | AX-170551355 | 7 | 43452596 | 4.54E-07 | -2.24 | 0.12 | None | NA |
| NuWe | | FarmCPU | AX-171476976 | 7 | 43476594 | 4.54E-07 | -2.24 | 0.12 | [glutamate receptor 3.6-like isoform X1 [Juglans regia]](https://blast.ncbi.nlm.nih.gov/Blast.cgi#alnHdr_1098809854) | 0.91 |
| KePe | | FarmCPU | AX-171127620 | 3 | 4593747 | 5.44E-07 | -4.81 | 0.11 | None | NA |
| KePe | | FarmCPU | AX-171127613 | 3 | 4607799 | 5.44E-07 | -4.81 | 0.11 | [probable mediator of RNA polymerase II transcription subunit 26b isoform X2 [Juglans regia]](https://blast.ncbi.nlm.nih.gov/Blast.cgi#alnHdr_1098809161) | 3e-09 |
| KePe | | FarmCPU | AX-171127592 | 3 | 4622487 | 5.44E-07 | -4.81 | 0.11 | [uncharacterized protein LOC108981537 [Juglans regia]](https://blast.ncbi.nlm.nih.gov/Blast.cgi#alnHdr_1098804817) | 1.00 |
| KePe | | FarmCPU | AX-171127578 | 3 | 4631072 | 5.44E-07 | -4.81 | 0.11 | [tubulin beta chain-like [Juglans regia]](https://blast.ncbi.nlm.nih.gov/Blast.cgi#alnHdr_1098845096) | 5e-11 |
| KePe | | FarmCPU | AX-171127550 | 3 | 4660678 | 5.44E-07 | -4.81 | 0.11 | None | NA |
| KePe | | FarmCPU | AX-171492963 | 3 | 4719248 | 5.44E-07 | -4.81 | 0.11 | [TMV resistance protein N-like [Juglans regia]](https://blast.ncbi.nlm.nih.gov/Blast.cgi#alnHdr_1098830651) | 2.2 |
| ShIn | | FarmCPU | AX-171182868 | 4 | 534843 | 1.30E-17 | 12.24 | 0.11 | [probable WRKY transcription factor 70 isoform X1 [Juglans regia]](https://blast.ncbi.nlm.nih.gov/Blast.cgi#alnHdr_1098825835) | 1e-12 |
| ShIn | | FarmCPU | AX-170782648 | 13 | 318671 | 3.31E-13 | 5.70 | 0.50 | None | NA |
| ShIn | | FarmCPU | AX-170630383 | 10 | 16819613 | 2.93E-11 | 4.62 | 0.32 | [LEAF RUST 10 DISEASE-RESISTANCE LOCUS RECEPTOR-LIKE PROTEIN KINASE-like 1.2 isoform X2 [Juglans regia]](https://blast.ncbi.nlm.nih.gov/Blast.cgi#alnHdr_1098851914) | 4.2 |
| ShIn | | FarmCPU | AX-171158510 | 3 | 280565 | 1.82E-08 | 3.95 | 0.4 | [WD repeat-containing protein 3 isoform X2 [Juglans regia]](https://blast.ncbi.nlm.nih.gov/Blast.cgi#alnHdr_1098752348) | 7e-09 |
| ShIn | | FarmCPU | AX-171164779 | 7 | 8899757 | 2.38E-07 | -5.72 | 0.07 | [acidic endochitinase-like [Juglans regia]](https://blast.ncbi.nlm.nih.gov/Blast.cgi#alnHdr_1098763877) | 5e-04 |
| ShIn | | FarmCPU | AX-170940898 | 10 | 6331821 | 2.64E-07 | 3.26 | 0.39 | [WD repeat-containing protein RUP2 [Juglans regia]](https://blast.ncbi.nlm.nih.gov/Blast.cgi#alnHdr_1098848036) | 4e-05 |
| ShIn_BLUP | | FarmCPU | AX-170782648 | 13 | 318671 | 1.12E-12 | 4.25 | 0.49 | None | NA |
| ShIn_BLUP | | FarmCPU | AX-171182868 | 4 | 534843 | 1.40E-10 | 6.86 | 0.11 | [probable WRKY transcription factor 70 isoform X1 [Juglans regia]](https://blast.ncbi.nlm.nih.gov/Blast.cgi#alnHdr_1098825835) | 1e-12 |
| RoIn | | FarmCPU | AX-170782648 | 13 | 318671 | 1.09E-15 | -0.041 | 0.49 | None | NA |
| RoIn | | FarmCPU | AX-170630383 | 10 | 16819613 | 1.25E-10 | -0.030 | 0.32 | [LEAF RUST 10 DISEASE-RESISTANCE LOCUS RECEPTOR-LIKE PROTEIN KINASE-like 1.2 isoform X2 [Juglans regia]](https://blast.ncbi.nlm.nih.gov/Blast.cgi#alnHdr_1098851914) | 4.2 |
| RoIn | | FarmCPU | AX-170963649 | 1 | 38525237 | 1.34E-10 | -0.060 | 0.06 | None | NA |
| RoIn | | FarmCPU | AX-171194911 | 10 | 25234618 | 7.05E-09 | -0.024 | 0.42 | None | NA |
| RoIn | | FarmCPU | AX-171182868 | 4 | 534843 | 4.60E-08 | -0.044 | 0.10 | [probable WRKY transcription factor 70 isoform X1 [Juglans regia]](https://blast.ncbi.nlm.nih.gov/Blast.cgi#alnHdr_1098825835) | 1e-12 |
| RoIn | | FarmCPU | AX-171542899 | 10 | 37308117 | 5.62E-08 | -0.027 | 0.26 | None | NA |
| RoIn | | FarmCPU | AX-171065124 | 8 | 26977340 | 9.27E-08 | -0.044 | 0.08 | None | NA |
| RoIn | | FarmCPU | AX-170940051 | 6 | 35597613 | 2.83E-07 | -0.026 | 0.26 | [uncharacterized protein LOC108994934 [Juglans regia]](https://blast.ncbi.nlm.nih.gov/Blast.cgi#alnHdr_1098838499) | 5.5 |
| RoIn | | FarmCPU | AX-171158510 | 3 | 280565 | 4.38E-07 | -0.024 | 0.4 | [WD repeat-containing protein 3 isoform X2 [Juglans regia]](https://blast.ncbi.nlm.nih.gov/Blast.cgi#alnHdr_1098752348) | 7e-09 |
| RoIn_BLUP | | FarmCPU | AX-170782648 | 13 | 318671 | 1.32E-10 | -0.021 | 0.49 | None | NA |
| RoIn_BLUP | | FarmCPU | AX-171065124 | 8 | 26977340 | 2.36E-10 | -0.038 | 0.08 | None | NA |
| RoIn_BLUP | | FarmCPU | AX-171511328 | 7 | 37269636 | 3.79E-10 | -0.031 | 0.09 | [BTB/POZ domain-containing protein At1g21780-like [Juglans regia]](https://blast.ncbi.nlm.nih.gov/Blast.cgi#alnHdr_1098803825) | 8.4 |
| RoIn_BLUP | | FarmCPU | AX-170963649 | 1 | 38525237 | 1.25E-09 | -0.040 | 0.06 | [probable plastid-lipid-associated protein 14, chloroplastic [Juglans regia]](https://blast.ncbi.nlm.nih.gov/Blast.cgi#alnHdr_1098782305) | 0.001 |
| RoIn_BLUP | | FarmCPU | AX-170932544 | 7 | 9801617 | 4.36E-09 | -0.020 | 0.28 | [LRR receptor-like serine/threonine-protein kinase GSO1 [Juglans regia]](https://blast.ncbi.nlm.nih.gov/Blast.cgi#alnHdr_1098842286) | 1e-11 |
| RoIn_BLUP | | FarmCPU | AX-171182868 | 4 | 534843 | 5.01E-08 | -0.031 | 0.11 | [probable WRKY transcription factor 70 isoform X1 [Juglans regia]](https://blast.ncbi.nlm.nih.gov/Blast.cgi#alnHdr_1098825835) | 1e-12 |
| PC1 | | FarmCPU | AX-170970503 | 3 | 13351304 | 7.76E-08 | -1.70 | 0.07 | [uncharacterized protein LOC108986101 [Juglans regia]](https://blast.ncbi.nlm.nih.gov/Blast.cgi#alnHdr_1098816053) | 2e-06 |
| PC1 | | FarmCPU | AX-170754307 | 2 | 1193372 | 9.91E-08 | -1.77 | 0.07 | None | NA |
| PC1 | | FarmCPU | AX-170969934 | 3 | 12888399 | 1.48E-07 | -1.19 | 0.13 | None | NA |
| PC2 | | FarmCPU | AX-171226096 | 7 | 28240772 | 2.79E-07 | -1.31 | 0.12 | None | NA |
| PC3 | | FarmCPU | AX-171147338 | 3 | 6729104 | 4.79E-07 | -1.68 | 0.13 | [uncharacterized protein LOC109021893 isoform X2 [Juglans regia]](https://blast.ncbi.nlm.nih.gov/Blast.cgi#alnHdr_1098797375) | 1.3 |
| PC3 | | FarmCPU | AX-171147330 | 3 | 6734531 | 4.79E-07 | -1.68 | 0.13 | [serine-threonine kinase receptor-associated protein-like [Juglans regia]](https://blast.ncbi.nlm.nih.gov/Blast.cgi#alnHdr_1098774851) | 1.6 |
| PC4 | | FarmCPU | AX-170827155 | 2 | 17563537 | 9.72E-15 | -1.81 | 0.06 | [uncharacterized protein LOC109003790 [Juglans regia]](https://blast.ncbi.nlm.nih.gov/Blast.cgi#alnHdr_1098720194) | 4e-05 |
| PC4 | | FarmCPU | AX-170863695 | 15 | 18913867 | 1.00E-14 | -1.51 | 0.06 | None | NA |
| PC4 | | FarmCPU | AX-170829132 | 9 | 15911669 | 2.84E-09 | -0.68 | 0.23 | [classical arabinogalactan protein 9-like [Juglans regia]](https://blast.ncbi.nlm.nih.gov/Blast.cgi#alnHdr_1098832281) | 1.1 |
| PC4 | | FarmCPU | AX-171167249 | 10 | 33271265 | 1.13E-08 | -0.85 | 0.07 | None | NA |
| PC4 | | FarmCPU | AX-170933380 | 7 | 49841622 | 2.42E-08 | -0.51 | 0.39 | [uncharacterized protein LOC109013465 [Juglans regia]](https://blast.ncbi.nlm.nih.gov/Blast.cgi#alnHdr_1098722869) | 3.5 |
| PC4 | | FarmCPU | AX-171127729 | 3 | 1091883 | 6.75E-08 | 0.49 | 0.49 | None | NA |
| PC4 | | FarmCPU | AX-170785842 | 6 | 12048693 | 2.69E-06 | 0.77 | 0.06 | [flavin-dependent oxidoreductase FOX2-like [Juglans regia]](https://blast.ncbi.nlm.nih.gov/Blast.cgi#alnHdr_1098804573) | 1.5 |
| PC5 | | FarmCPU | AX-171078457 | 15 | 15213903 | 4.95E-13 | -1.83 | 0.05 | [40S ribosomal protein S7-like [Juglans regia]](https://blast.ncbi.nlm.nih.gov/Blast.cgi#alnHdr_1098821204) | 9.2 |
| PC5 | | FarmCPU | AX-170866883 | 2 | 33416492 | 2.72E-10 | -1.04 | 0.11 | None | NA |
| PC5 | | FarmCPU | AX-171044455 | 8 | 11317330 | 2.45E-07 | -0.62 | 0.15 | [uncharacterized protein LOC108980548 [Juglans regia]](https://blast.ncbi.nlm.nih.gov/Blast.cgi#alnHdr_1098802421) | 8e-07 |
| PC5 | | FarmCPU | AX-171205325 | 1 | 37693125 | 3.56E-07 | -0.51 | 0.26 | [arginine--tRNA ligase, chloroplastic/mitochondrial-like isoform X2 [Juglans regia]](https://blast.ncbi.nlm.nih.gov/Blast.cgi#alnHdr_1098741917) | 1e-08 |
| PC5 | | FarmCPU | AX-170966043 | 4 | 10953309 | 3.63E-07 | -0.83 | 0.06 | None | NA |
| PC5 | | SUPER | AX-171078457 | 15 | 15213903 | 1.18E-13 | -1.83 | 0.05 | [40S ribosomal protein S7-like [Juglans regia]](https://blast.ncbi.nlm.nih.gov/Blast.cgi#alnHdr_1098821204) | 9.2 |
|  | Annotations of suggestive SNPs for nut and kernel related traits | | | | | | | | | |
| NuLe | | FarmCPU | AX-171523208 | 13 | 522227 | 4.50E-06 | 2.53 | 0.49 | None | NA |
| NuLe | | FarmCPU | AX-171523218 | 13 | 491366 | 6.80E-06 | 2.49 | 0.4 | None | NA |
| NuLe | | FarmCPU | AX-170782452 | 13 | 495465 | 6.80E-06 | 2.49 | 0.4 | None | NA |
| NuLe | | FarmCPU | AX-170589187 | 10 | 36956153 | 6.84E-06 | 3.04 | 0.16 | None | NA |
| SiIn | | FarmCPU | AX-170990439 | 1 | 8914651 | 3.85E-06 | -1.92 | 0.26 | None | NA |
| SiIn | | FarmCPU | AX-170990851 | 1 | 8554385 | 6.97E-06 | -1.94 | 0.27 | None | NA |
| SiIn | | FarmCPU | AX-171563772 | 1 | 8552151 | 9.76E-06 | -1.93 | 0.27 | None | NA |
| SiIn | | FarmCPU | AX-170697627 | 7 | 42230537 | 9.97E-06 | 2.30 | 0.15 | [putative receptor-like protein kinase At4g00960 [Juglans regia]](https://blast.ncbi.nlm.nih.gov/Blast.cgi#alnHdr_1098801482) | 1.5 |
| NuSh | | FarmCPU | AX-171105437 | 4 | 6315247 | 1.07E-05 | -1.16 | 0.48 | [protein FAR1-RELATED SEQUENCE 5-like [Juglans regia]](https://blast.ncbi.nlm.nih.gov/Blast.cgi#alnHdr_1098766305) | 1.5 |
| NuSh | | FarmCPU | AX-171105430 | 4 | 6326737 | 1.07E-05 | -1.16 | 0.48 | [fatty acid amide hydrolase isoform X1 [Juglans regia]](https://blast.ncbi.nlm.nih.gov/Blast.cgi#alnHdr_1098796730) | 0.004 |
| SheTh_BLU | | FarmCPU | AX-171094681 | 13 | 15556476 | 1.08E-05 | -0.23 | 0.19 | None | NA |
| SheCo | | FarmCPU | AX-170688173 | 2 | 24388882 | 4.67E-06 | -1.60 | 0.09 | None | NA |
| SheCo | | FarmCPU | AX-171504445 | 2 | 24406393 | 4.67E-06 | -1.60 | 0.09 | None | NA |
| SheCo | | FarmCPU | AX-171581634 | 4 | 33988939 | 6.66E-06 | -1.02 | 0.45 | None | NA |
| SheCo | | FarmCPU | AX-171528376 | 4 | 33698720 | 7.79E-06 | -0.98 | 0.49 | None | NA |
| SheTe | | FarmCPU | AX-170775871 | 13 | 23087905 | 6.63E-06 | 2.44 | 0.07 | None | NA |
| SheTe | | FarmCPU | AX-171571438 | 7 | 7097384 | 7.57E-06 | 1.23 | 0.4 | None | NA |
| SheTe | | FarmCPU | AX-170756010 | 7 | 7028426 | 8.46E-06 | 1.21 | 0.39 | [uncharacterized protein LOC108984200 [Juglans regia]](https://blast.ncbi.nlm.nih.gov/Blast.cgi#alnHdr_1098811150) | 5e-05 |
| SheTe | | FarmCPU | AX-171571436 | 7 | 7102249 | 8.46E-06 | 1.21 | 0.39 | None | NA |
| SheTe | | FarmCPU | AX-171029639 | 7 | 7112592 | 8.46E-06 | 1.21 | 0.39 | None | NA |
| SheTe | | FarmCPU | AX-171205968 | 7 | 7120341 | 8.46E-06 | 1.21 | 0.39 | None | NA |
| SheTe | | FarmCPU | AX-171029629 | 7 | 7125087 | 8.46E-06 | 1.21 | 0.39 | [cysteine-rich receptor-like protein kinase 25 [Juglans regia]](https://blast.ncbi.nlm.nih.gov/Blast.cgi#alnHdr_1098786047) | 7e-04 |
| SheSe | | FarmCPU | AX-171515312 | 13 | 28819498 | 1.85E-06 | 0.99 | 0.18 | None | NA |
| SheSe | | FarmCPU | AX-170742415 | 13 | 28802576 | 2.41E-06 | 0.84 | 0.4 | [uncharacterized protein LOC108987318 [Juglans regia]](https://blast.ncbi.nlm.nih.gov/Blast.cgi#alnHdr_1098819156) | 2.6 |
| SheSe | | FarmCPU | AX-170742431 | 13 | 28780531 | 3.24E-06 | 0.85 | 0.41 | None | NA |
| SheSe | | FarmCPU | AX-170742430 | 13 | 28780580 | 3.33E-06 | 1.18 | 0.15 | [photosystem I reaction center subunit II, chloroplastic-like [Juglans regia]](https://blast.ncbi.nlm.nih.gov/Blast.cgi#alnHdr_1098767356) | 2.1 |
| SheSe | | FarmCPU | AX-170742419 | 13 | 28799809 | 3.33E-06 | 1.18 | 0.15 | None | NA |
| SheSe | | FarmCPU | AX-170860542 | 14 | 9148964 | 3.49E-06 | 1.48 | 0.08 | [uncharacterized protein LOC108993358 [Juglans regia]](https://blast.ncbi.nlm.nih.gov/Blast.cgi#alnHdr_1098834475) | 9.4 |
| SheSe | | FarmCPU | AX-171542846 | 13 | 28900918 | 5.54E-06 | 1.09 | 0.13 | None | NA |
| SheSt | | FarmCPU | AX-170562566 | 4 | 18065453 | 2.17E-06 | -0.93 | 0.27 | [pentatricopeptide repeat-containing protein At5g12100, mitochondrial [Juglans regia]](https://blast.ncbi.nlm.nih.gov/Blast.cgi#alnHdr_1098812597) | 2.6 |
| SheSt | | FarmCPU | AX-171146058 | 3 | 832254 | 3.46E-06 | 0.91 | 0.41 | None | NA |
| KeWe | | FarmCPU | AX-171123624 | 12 | 28982617 | 8.08E-06 | -0.70 | 0.31 | [NAC domain-containing protein 43-like [Juglans regia]](https://blast.ncbi.nlm.nih.gov/Blast.cgi#alnHdr_1098806126) | 8.5 |
| KeWe | | FarmCPU | AX-171070005 | 13 | 26610897 | 8.62E-06 | -0.66 | 0.4 | [vacuolar protein sorting-associated protein 36 [Juglans regia]](https://blast.ncbi.nlm.nih.gov/Blast.cgi#alnHdr_1098795013) | 1.4 |
| KeWe | | FarmCPU | AX-171069969 | 13 | 26634916 | 8.62E-06 | -0.66 | 0.4 | None | NA |
| KeWe | | FarmCPU | AX-171069989 | 13 | 26618317 | 9.39E-06 | -0.65 | 0.41 | [sorting nexin 2B-like [Juglans regia]](https://blast.ncbi.nlm.nih.gov/Blast.cgi#alnHdr_1098843567) | 4.7 |
| KeWe | | FarmCPU | AX-171069985 | 13 | 26618986 | 9.39E-06 | -0.65 | 0.41 | None | NA |
| KeCo | | FarmCPU | AX-170935503 | 10 | 19066386 | 2.72E-06 | -0.21 | 0.23 | None | NA |
| KeCo | | FarmCPU | AX-170935509 | 10 | 19073464 | 2.72E-06 | -0.21 | 0.23 | None | NA |
| KeCo | | FarmCPU | AX-170935534 | 10 | 19089621 | 2.72E-06 | -0.21 | 0.23 | None | NA |
| KeCo | | FarmCPU | AX-171135896 | 12 | 29052593 | 7.82E-06 | 0.99 | 0.34 | [uncharacterized protein LOC108984030 [Juglans regia]](https://blast.ncbi.nlm.nih.gov/Blast.cgi#alnHdr_1098810733) | 1e-08 |
| KePI | | FarmCPU | AX-171540153 | 6 | 596228 | 3.60E-06 | -1.23 | 0.07 | [glutamate receptor 2.1-like [Juglans regia]](https://blast.ncbi.nlm.nih.gov/Blast.cgi#alnHdr_1098748805) | 7.3 |
| KePI | | FarmCPU | AX-170869861 | 6 | 602708 | 3.60E-06 | -1.23 | 0.07 | None | NA |
| KePI | | FarmCPU | AX-171540148 | 6 | 604909 | 3.60E-06 | -1.23 | 0.07 | None | NA |
| KePI | | FarmCPU | AX-170798920 | 16 | 19031073 | 6.32E-06 | -0.84 | 0.17 | None | NA |
| KePI | | FarmCPU | AX-170799089 | 16 | 19174355 | 1.09E-05 | -0.78 | 0.21 | None | NA |
| KeFi | | FarmCPU | AX-171522879 | 13 | 12279892 | 6.32E-06 | -0.85 | 0.10 | None | NA |
| KeFi | | FarmCPU | AX-171180908 | 13 | 12197515 | 9.65E-06 | -0.89 | 0.09 | [uncharacterized protein LOC108988788 [Juglans regia]](https://blast.ncbi.nlm.nih.gov/Blast.cgi#alnHdr_1098822841) | 1e-09 |
| KeFi | | FarmCPU | AX-171212523 | 13 | 11832504 | 1.02E-05 | -0.92 | 0.11 | [cyclic dof factor 2-like [Juglans regia]](https://blast.ncbi.nlm.nih.gov/Blast.cgi#alnHdr_1098803192) | 1e-08 |
| KeFi_BLU | | FarmCPU | AX-171034205 | 8 | 10403102 | 2.00E-06 | 0.29 | 0.28 | None | NA |
| KeFi_BLU | | FarmCPU | AX-171591875 | 8 | 10391448 | 3.06E-06 | 0.33 | 0.24 | None | NA |
| KeFi_BLU | | FarmCPU | AX-170636004 | 8 | 10404157 | 3.51E-06 | 0.27 | 0.29 | None | NA |
| EKeNu | | FarmCPU | AX-171533041 | 7 | 51222580 | 1.88E-06 | 1.09 | 0.15 | [DNA mismatch repair protein MSH7 isoform X1 [Juglans regia]](https://blast.ncbi.nlm.nih.gov/Blast.cgi#alnHdr_1098832617) | 0.47 |
| EKeNu | | FarmCPU | AX-171506020 | 11 | 2373208 | 4.68E-06 | -0.90 | 0.27 | [zinc finger CCCH domain-containing protein 6-like isoform X2 [Juglans regia]](https://blast.ncbi.nlm.nih.gov/Blast.cgi#alnHdr_1098799438) | 2.6 |
| EKeNu | | FarmCPU | AX-171533025 | 7 | 51157854 | 5.26E-06 | 1.04 | 0.14 | None | NA |
| EKeNu | | FarmCPU | AX-171185813 | 7 | 51162248 | 7.64E-06 | 1.06 | 0.13 | None | NA |
| EKeNu | | FarmCPU | AX-171533023 | 7 | 51151297 | 9.63E-06 | 1.04 | 0.13 | None | NA |

MAF, Minor allele frequency.
Allelic effect, difference in mean of measured traits between genotypes with major allele and minor allele. Positive and negative signs indicates that the major allele is associated with increased and reduced measured traits respectively.
